# Covering all the Bases: Preclinical Development of an Effective *Staphylococcus aureus* Vaccine

**DOI:** 10.3389/fimmu.2014.00109

**Published:** 2014-03-24

**Authors:** Ingrid L. Scully, Paul A. Liberator, Kathrin U. Jansen, Annaliesa S. Anderson

**Affiliations:** ^1^Pfizer Vaccine Research and Development Unit, Pearl River, NY, USA

**Keywords:** vaccine, *Staphylococcus aureus*, clumping factor A, preclinical models, immunoassays, capsular polysaccharide conjugates, manganese transport protein C

## Abstract

A key aspect of the pathogenesis of the Gram positive bacterium *Staphylococcus aureus* is its ability to rapidly adapt to the host environment during the course of an infection. To successfully establish infection, the organism deploys a variety of survival and immune evasion strategies, ranging from the acquisition of essential nutrients and expression of adhesins, which promote colonization and survival, to the elaboration of virulence factors such as capsule, which aids host immune evasion. The ability of *S. aureus* to deploy different virulence factors must be taken into account for *S. aureus* vaccine design. Here, we present a strategy for designing an effective vaccine against *S. aureus* disease by evaluating vaccine candidate performance in multiple *in vivo* models targeted to mimic aspects of human disease, and by co-development of functional *in vitro* immunoassays that measure the neutralization of relevant *S. aureus* virulence factors.

## Introduction

One of the major challenges in developing a vaccine for *Staphylococcus aureus* is the organism’s ability to rapidly adapt to the host microenvironment. The Gram positive organism *S. aureus* is notorious for its diverse array of virulence factors, adaptation, and potential to change its surface antigen repertoire quickly (1).

The medical need for an *S. aureus* vaccine is clear. *S. aureus* is carried asymptomatically in the nares of 20–50% of the general population (2). *S. aureus* causes a wide spectrum of disease, ranging from relatively mild skin infections, such as carbuncles, to life-threatening wound and bloodstream infections (3). *S. aureus* is recognized as a leading cause of morbidity and mortality in both healthcare-associated and community settings. In surgical patients in particular, these infections carry high mortality rates; survivors of *S. aureus* surgical infections require an additional 13–17 days in the hospital, significantly increasing healthcare costs (4). The burden of *S. aureus* disease is exacerbated by antibiotic-resistant *S. aureus* isolates, highlighting the need for an effective prophylactic vaccine.

Specialized host microenvironments present obstacles for *S. aureus* to establish a productive infection. For example, in the blood, the organism must avoid ingestion and killing by phagocytes, especially neutrophils. In contrast, in a surgically induced wound, there are fewer neutrophils, at least initially, but nutrients are extremely limited and *S. aureu*s must adhere to host surfaces. *S. aureus* possesses a variety of means to both colonize the host and evade the immune system by inhibiting phagocytic uptake and killing. The bacterium has developed multiple additional virulence factors to enable it to adapt and survive in a variety of host niches and as a result, to cause a multitude of diverse infection pathologies. To establish and sustain infection in the host, *S. aureus* needs to:

- Find a means of entry into the host by breaching the protective physical barriers to infection (skin, mucosa), or by entering a lesion, such as those created during surgery or traumatic injury.
- Obtain the essential nutrients for growth that are restricted in the host, such as iron and manganese, by expressing iron acquisition receptors, such as IsdB (5), and manganese uptake receptors, such as the ABC transporter MntABC (6).
- Adhere to extracellular matrix components and host cells. *S. aureus* can express multiple adhesion factors, including fibronectin-binding proteins (FnBPA, FnBPB) (7, 8), fibrinogen-binding proteins, also known as “clumping factors” (ClfA, ClfB) (9, 10), and collagen-binding protein (Cna) (11).
- Evade immune killing by producing an anti-phagocytic polysaccharide capsule (CP5 or CP8) (12) and elaborating a number of proteins that can interfere with antibody and complement binding (SpA, Sbi, SCIN) (13–15), phagocytic uptake, and killing in the phagolysosome. In addition, *S. aureus* can evade immune killing by producing multiple toxins that are either lethal to immune cells, such as alpha toxin and Panton–Valentine leukocidin (PVL), or act as superantigens, such as toxic shock syndrome toxin (TSST) and staphylococcal enterotoxin B (SEB) (16–18).

These different mechanisms of pathogenesis render *S. aureus* a challenging vaccine target. Thus, an effective vaccine to prevent *S. aureus* disease must contain antigens carefully selected to interrupt *S. aureus* pathogenesis using a multipronged approach. Currently there is no licensed vaccine against *S. aureus* infection and no clinically demonstrated correlate of protection against *S. aureus*.

## Immune Defenses Against *S. aureus* Infection

The human host has several mechanisms to prevent *S. aureus* infection. These include mucosal and epithelial layers that act as a barrier to infection, the sequestration of essential nutrients to prevent bacterial survival and replication, the elaboration of danger signals in response to microbial products to activate the innate immune response and engulfment and killing of bacteria by professional phagocytes, such as neutrophils. In addition, adaptive immune components such as antibodies and T cells can act to prevent infection.

Humans can be persistently colonized with *S. aureus* and as such, there can be a constant interaction between the bacteria and the host immune system. This is supported by the observation that all adults have pre-existing binding antibodies to *S. aureus* antigens, including capsule and ClfA (19). However, functional antibodies that facilitate the opsonophagocytic killing of staphylococci by professional phagocytes [particularly neutrophils (20, 21)] and functional antibodies that neutralize virulence factors are absent in the vast majority of the human population. The importance of bacterial killing to prevent and/or resolve infections is underscored by the increased rates of infection that are observed for subjects with immunological disorders. The close link between defects in clearance and the risk of disease supports the hypothesis that if a vaccine generates adequate levels of functional antibodies in subjects with competent effector cells, these individuals are more likely to be protected at times of risk for an infection.

Polymorphonuclear neutrophils (PMNs) are the primary cellular defense against staphylococcal infections. These effector cells are highly efficient at killing phagocytosed pathogens. Phagocytosis is the binding and ingestion of bacteria, which can be facilitated by opsonization of the microbial surfaces with antibody and/or complement (22). Neutrophils recognize many molecules produced by *S. aureus*, such as lipoteichoic acid (LTA) and CpG DNA, which interact with TLRs on the neutrophils to promote phagocytosis (23, 24). Neutrophils express on their cell surfaces both complement receptors that bind complement-opsonized bacteria, and Fc receptors that bind the Fc region of antibody-coated bacteria. Once bound to the neutrophils, the bacteria are engulfed into an intracellular phagosome. This is followed by neutrophil-initiated mechanisms to kill the bacteria within the phagosome. Phagocytosis activates membrane bound NADPH-dependent oxidase to initiate a “respiratory burst,” which generates high levels of reactive oxygen species (ROS, e.g., superoxide radicals, hydrogen peroxide, and hydroxyl radicals) to kill the engulfed microorganisms. In addition, the fusion of cytoplasmic granules with the bacteria-containing phagosome results in the release of pre-formed anti-microbial peptides, proteases, and α-defensins with potent microbicidal activity (25).

Individuals with PMN defects have a higher incidence of *S. aureus* disease. Examples of neutrophil dysfunction include frank neutropenia (26), chronic granulomatous disease, in which neutrophils are unable to generate a functional respiratory burst (27–29), and Chediak–Higashi Syndrome, in which individuals have neutrophils with reduced chemotaxis and phagolysosome function (30). Subjects with defects in non-antibody-mediated clearance mechanisms, such as those with mutations in the pattern recognition receptor TLR2 (31) or defects in the complement pathway (32) cannot effectively clear staphylococci and thus are more susceptible to *S. aureus* infections. Individuals with defective STAT3 signaling proteins have also been shown to be more prone to *S. aureus* infections due to the inability to generate IL-17-producing Th17 cells, which results in diminished neutrophil recruitment and function (33–35).

Antibody-mediated clearance mechanisms driven by the adaptive immune system, in which antibody-secreting B cells and cytokine-secreting and cytolytic T cells play a key role, are also important in the prevention of staphylococcal disease. For example, individuals with immune defects impairing the ability to produce functional antibodies, such as acquired immune deficiency syndrome (36), immature immune systems due to premature birth (37), or defects in immunoglobulin production, all have increased susceptibility to staphylococcal infections (38, 39). In males with X-linked agammaglobulinemia, recurrent pyogenic infections that are often caused by *S. aureus* begin to occur within the first year of life after maternal IgG has been exhausted (40). Therefore, an effective *S. aureus* vaccine will likely require that a functional antibody response is elicited to enhance phagocyte-mediated killing of the organism. Functional antibody responses require the concomitant development of T cell help provided by CD4^+^ T cells. In addition, cytokines secreted by T helper cells (both Th1 and Th17) have been shown to enhance neutrophil effector function, so vaccine-induced Th1 and/or Th17 responses may also be beneficial in preventing *S. aureus*-mediated disease (41). Overall, these findings support the requirement for functional antibodies and neutrophil effector cell function to prevent *S. aureus* infections and disease.

However, *S. aureus* has virulence mechanisms that counteract host defenses to enable establishment of invasive *S. aureus* (ISA) disease. These mechanisms include scavenging essential nutrients, adhering to host tissues, and immune evasion (42). Therefore, for a vaccine to prevent ISA, these mechanisms also need to be taken into consideration to prevent the organism from having the opportunity to establish a productive infection.

## Previous Vaccine Approaches and Why They may Not have been Successful

Previous prophylactic *S. aureus* vaccines have failed in the clinic; these vaccines were composed of single antigens, either a bivalent capsular polysaccharide formulation or IsdB, and thus could only target a single virulence mechanism. Capsular polysaccharides help bacteria evade immune-mediated killing through inhibiting phagocytosis (12, 43). Vaccine-induced antibodies against capsular polysaccharides can overcome this virulence mechanism, but do not address other virulence mechanisms, potentially limiting the effectiveness of capsule-alone vaccine approaches. Capsular polysaccharide conjugate vaccines, such as Prevnar-13, are capable of inducing functional opsonophagocytic killing responses. The capsular polysaccharide conjugate vaccine StaphVAX did show a 57% decrease (compared to control) in incidence of bacteremia in its initial phase 3 efficacy study in hemodialysis patients. This protective effect lasted up to 40 weeks post-vaccination (44). Subsequently, in a larger phase 3 study of StaphVAX, no significant protection was observed (45). The results with StaphVAX sharply contrast the striking success of capsular polysaccharide-based vaccines in protecting against disease caused by *Haemophilus influenzae* type B (HiB) (46, 47), *Neisseria meningitidis* serogroup A, C, Y, and W-135 (48–50) and *Streptococcus pneumoniae* (51, 52). There were many potential reasons why the StaphVAX pivotal trial failed, including the selection of the phase 3 clinical population (end stage renal disease subjects), which has a high incidence of disease but is also considered to be somewhat immunosuppressed. In addition, there may have been issues with consistency of manufacture of the phase 3 clinical trial material and the evidence that functional bacterial killing responses were elicited was weak (45). However, even if potent bacterial killing responses would have been induced, a capsular polysaccharide conjugate-alone approach may still not have been sufficient to confer protection against *S. aureus* disease due to the multiple virulence mechanisms possessed by *S. aureus*.

Likewise, a vaccine targeting IsdB may inhibit *S. aureus* acquisition of the essential nutrient iron, but it does not impact anti-phagocytic virulence mechanisms. This may help explain why V710, a prophylactic monovalent vaccine composed of IsdB (53) and developed to prevent *S. aureus* infections in patients undergoing cardiothoracic surgery, failed to show significant efficacy in preventing *S. aureus* infections. A recent publication has shown that adults do generate titers to IsdB through natural exposure, and titers approximately double in hospitalized patients with *S. aureus* infections, but not other patients, indicating that IsdB is expressed during infection (54). However, the V710 IsdB vaccine does not appear to induce antibodies that efficiently induce killing of live *S. aureus* bacteria. Instead, antibodies induced against IsdB have only been shown to facilitate the uptake of *S. aureus* cells into neutrophils. None of the available clinical data of the V710 IsdB vaccine describe or document whether robust functional *S. aureus* killing responses were induced in the vaccine trials (55) or pre-clinically (56), with the exception of some data that individual mAbs recognizing IsdB (the vaccine’s single antigen target) did show functional killing activity (57, 58). IsdB is generally expressed relatively late in infection in preclinical models (59), but did show some protection in lethal challenge models (60, 61). Stranger-Jones et al. demonstrated that the addition of IsdA, SdrD, and SdrE converted partial protection for each individual antigen to complete protection in a lethal challenge model, highlighting the potential benefits of a multiantigen approach (62). Thus, vaccines targeting a single antigen or phase of pathogenesis or infection type are not likely to be broadly protective and a multiantigen approach targeting multiple virulence mechanisms is likely required to protect against *S. aureus* infection and disease.

## Multiantigen Vaccine Approaches

Given the low likelihood of success with a single antigen approach, Pfizer and others are currently developing multiantigen investigational *S. aureus* vaccines. Pfizer’s four antigen investigational *S. aureus* vaccine (SA4Ag) is comprised of the microbial surface components recognizing adhesive matrix molecules (MSCRAMM) ClfA, the manganese transporter component MntC (aka rP305A or SA0688), and capsular polysaccharides type 5 and 8. Each antigen was carefully selected to block *S. aureus* virulence mechanisms involved in the establishment and/or maintenance of infection.

To establish ISA disease, the pathogen must first adhere to host cells, extracellular matrix, or an implanted medical device. Staphylococcal clumping factor A (ClfA), originally identified by Foster and colleagues (63), is a member of the MSCRAMM family of bacterial adhesion molecules. ClfA derives its name from its ability to induce platelet aggregation, or clumping. ClfA specifically binds to the C-terminal end of the fibrinogen γ-chain. ClfA is the major fibrinogen-binding protein in *S. aureus* (9, 64), and as such plays an important role in establishing wound and foreign body infections. Foster and colleagues elucidated that ClfA is essential during the early stages of infection and its importance as a *S. aureus* virulence factor has been demonstrated in several small animal models of infection, including endocarditis, arthritis, and sepsis (65–67). Interrupting ClfA binding to fibrinogen through the generation of inhibitory antibodies induced by vaccination may be beneficial in preventing the establishment of infection.

Once an infection is established, *S. aureus* must acquire essential nutrients for survival in the host microenvironment. One essential nutrient is manganese, which *S. aureus* requires for normal cell metabolism. Manganese is also a cofactor for the enzyme superoxide dismutase (SOD), which is critical for detoxifying oxygen radicals, such as those released during the bactericidal neutrophil respiratory burst. *S. aureus* acquires manganese via the ABC transporter complex MntABC. MntC is the metal-binding component of the complex, and is a surface-exposed lipoprotein. Indeed, recent studies have shown that MntC-deficient *S. aureus* is more sensitive to reactive oxygen species (68). Although MntC is poorly expressed *in vitro*, it is rapidly expressed in the host microenvironment (59) and in biofilms (69). In a study of *S. aureus* bacteremic patients, the median-fold increase in antibody titer against MntC from initial infection to peak titer was 5.17, the highest of the 56 gene products surveyed. In addition, the same study showed that all 21 isolates evaluated possessed the *mntC* gene (70). These characteristics make MntC an attractive vaccine target.

Finally, once *S. aureus* has invaded the host, it must avoid elimination by the host immune system. *S. aureus* has many mechanisms for immune evasion, including complement-binding proteins, immunoglobulin-binding proteins, such as protein A, and leukocidins such as alpha toxin, which kill white blood cells. In addition, *S. aureus* elaborates capsular polysaccharide, which has anti-phagocytic properties. All invasive human *S. aureus* have the genes required to express either type 5 or 8 capsule (denoted CP5 and CP8, respectively), and most adults have capsular-binding antibodies, demonstrating that the capsules are expressed *in vivo*. Due to its highly repetitive nature, capsular antigens have high epitope density and thus are attractive candidates for prophylactic vaccines.

In addition to Pfizer’s SA4Ag approach, others are also developing multiantigen vaccines targeting *S. aureus*, composed of capsular polysaccharide conjugates and/or protein antigens. Some vaccine approaches include the addition of toxins, many of which have shown preclinical efficacy in specific animal models, such as alpha toxin, PVL, SEB, and TSST (71–73). However, many toxins, including PVL, TSST, and SEB, are only expressed by a proportion of strains (74, 75), limiting the potential coverage afforded by these vaccine candidates. In contrast, some toxins, such as alpha toxin, also known as α-hemolysin, are genetically conserved and thus have the potential to protect against a wide variety of *S. aureus* strains. Alpha toxin is a pore-forming β-barrel toxin, which binds to host cell receptors such as ADAM10 to initiate toxin oligomerization and pore formation. High levels of alpha toxin expression have been correlated with poor outcomes in pneumonia models. Alpha toxin has also been implicated in *S. aureus* pathogenesis in dermonecrosis and bacteremia models. Antibody therapeutics directed against alpha toxin have been shown to be effective in preclinical models (76, 77), so alpha toxin may be a beneficial addition to multiantigen *S. aureus* vaccines. Antitoxin approaches in general, though, are challenged by a number of factors: (1) the sheer number and redundancy of toxins produced by *S. aureus* (over 50) make selection of toxins for vaccine inclusion difficult, (2) toxins are generally produced relatively late in the infectious process, so antitoxin responses will have an effect later in the infectious cycle, after disease has been established, and (3) toxins are generally secreted factors, and thus antitoxin responses will not directly lead to killing and clearance of *S. aureus*.

The addition of an adjuvant may be beneficial, depending on the antigens selected, the desired immune response, and the target population(s) intended for vaccination. For example, if the intended population is naïve to *S. aureus* exposure or immunocompromised, addition of an adjuvant may boost immune responses. Some antigens, such as polysaccharide conjugates, generally do not require the addition of an adjuvant, while others, such as poorly immunogenic proteins, may greatly benefit from adjuvantation, even in immunocompetent subjects. Adjuvants can drive a more cell-mediated biased immune response, a more humoral biased immune response, or a balanced response, given the intrinsic properties of the adjuvant. However, the optimal adjuvant to use with a particular antigen or set of antigens or for a particular target population ultimately must be empirically determined.

## Development of Preclinical Animal Models and *In vitro* Assays to Assess Vaccine Function

Both careful selection of preclinical animal models and development of *in vitro* assays that measure functional serological responses are important for effective vaccine design.

*S. aureus* causes a wide range of disease in a variety of host microenvironments, so the preclinical development of an effective vaccine targeting *S. aureus* must involve the use of multiple preclinical *in vivo* models, which represent different diseases. Each model selected provides information on how the vaccine might behave clinically. In the case of *S. aureus*, end organ dissemination can be mimicked by pyelonephritis models, the response to deep tissue infections can be approximated by wound models, and bloodstream infections can be modeled by bacteremia and/or sepsis models. When selecting preclinical animal models, it is important that the model replicates salient features of the vaccine target population. For example, if the vaccine is aimed at preventing *S. aureus* pneumonia, then a pneumonia model should be evaluated pre-clinically. Ideally, non-human primate models are used, but non-human primate models are expensive and numerically limited. Small animal models have been criticized, especially in the *S. aureus* vaccine field, for not accurately predicting responses in the clinic. While it is true that all models have limitations, when used with discretion they can provide important insights into pathogenic mechanisms, host–pathogen interactions, and kinetics of antigen expression *in vivo*. These insights can guide vaccine antigen selection to ensure that a vaccine is broadly protective against a range of *S. aureus* disease manifestations. In the case of StaphVAX, if the new phase 3 lots for the second phase 3 trial had been tested in either preclinical models or in potency-indicating studies, an expensive and unsuccessful efficacy study might have been avoided.

Equally as important as the demonstration that an investigational *S. aureus* vaccine elicits a protective response in multiple animal models that mimic different types of *S. aureus* infection, is the use of diverse *S. aureus* isolates in preclinical models. Disease-causing *S. aureus* are genetically diverse, and thus vaccine-induced immune responses might have varying effectiveness against genetically distinct clinical isolates. For example, disease-causing *S. aureus* possesses the genetic machinery to produce one of two capsular polysaccharide types, type 5 or 8. Immune responses against CP5 may or may not cross-protect against CP8 isolates, and vice versa, so clinical strains expressing each capsule type need to be tested in animal models.

In addition to developing a battery of preclinical animal models that can be used to evaluate vaccine efficacy against a variety of *S. aureus* disease manifestations, it is also important to concomitantly develop *in vitro* assays that measure development of functional immune responses from both preclinical and clinical specimens. Assays that measure functional immune responses are preferable to assays that merely measure antigen-binding antibody, such as ELISAs. For example, functional killing antibody responses against *S. aureus* capsular polysaccharide can be detected using opsonophagocytic activity assays (OPAs). We have previously shown that *S. aureus* CP–CRM_197_ conjugates induce robust killing OPA responses (12), and we and others have shown that capsular polysaccharide is an important immune evasion mechanism. Thus, OPA titers can be used as another measure potential protection against *S. aureus* disease.

In the case of protein antigens, an assay which measures interruption of protein function can be desirable. Functional immune responses against the *S. aureus* adhesion molecule ClfA can be measured by means of an adhesion assay using live bacteria, the fibrinogen-binding inhibition (FBI) assay. The FBI quantitates the ability of immune serum to inhibit binding of whole live *S. aureus* to fibrinogen, which occurs through the inhibition of ClfA binding to its cognate ligand. The FBI assay has been used to demonstrate that while unimmunized subjects have pre-existing high-binding antibody titers to ClfA, only very few individuals have functional antibodies that can abrogate the binding of ClfA to fibrinogen (19). For protein antigens, there is potential genetic diversity, so isolates containing disparate alleles should be tested. In addition to testing isolates that are diverse at the vaccine antigen level, it is also prudent to test isolates from diverse disease-associated clonal complexes. By including multiple clinical isolates that are genetically distinct at the antigen level as well as surveying the universe of disease-causing clonal complexes, a better sense of the possible breadth of coverage for an investigational *S. aureus* vaccine can be obtained.

The development of functional assays to assess immune responses to vaccine antigens yields greater insight into the quality of immune responses elicited by an investigational vaccine. Such functional assays can generally be used to measure both preclinical and clinical immune responses. Thus target values for vaccine-induced responses can be set, based on protective thresholds identified in preclinical animal models. If subsequently verified in the clinic, functional immunological assays can become the basis of an immune correlate of protection.

As described above, employing a wholistic, multiparameter approach to preclinical evaluation of *S. aureus* vaccine candidate antigens may be critical for development of an effective *S. aureus* vaccine. Due to the versatility of *S. aureus* as it adapts to host microenvironments, multiple preclinical models must be used which mimic aspects of human *S. aureus* disease. Likewise, protection needs to be demonstrated against a number of diverse *S. aureus* isolates. A successful vaccine against *S. aureus* will likely need to counter multiple virulence mechanisms, such as initial adhesion events, nutrient acquisition, and immune evasion. Monitoring efficacy of candidate antigens in multiple preclinical models provides an opportunity to understand the strengths and weaknesses of candidate antigens, and to select a set of vaccine candidates, which complement each other. The concomitant development of *in vitro* assays, such as the OPA, to monitor functional immune responses enables benchmarking of observations made in preclinical *in vivo* models with those made in the clinic. The linking of a diversity of preclinical animal models with functional *in vitro* assays will likely improve the probability of developing a broadly efficacious *S. aureus* vaccine.

## Conflict of Interest Statement

Ingrid L. Scully, Paul A. Liberator, Kathrin U. Jansen, and Annaliesa S. Anderson are employes of Pfizer and as such may hold stock in the company.
